# Case Report: Clinical characteristics and multidisciplinary collaborative management of congenital tuberculosis in extremely preterm twins and preterm infants: a report of three cases and literature review

**DOI:** 10.3389/fped.2025.1674447

**Published:** 2026-01-15

**Authors:** Liming Yang, Lunli Yue, Qiong Chen, Xi Huang

**Affiliations:** 1 Department of Neonatology Nursing, West China Second University Hospital, Sichuan University, Chengdu, China; 2 Key Laboratory of Birth Defects and Related Diseases of Women and Children, Sichuan University, Ministry of Education, Chengdu, China

**Keywords:** anti-tuberculosis treatment, congenital tuberculosis, fundus screening, maternal-fetal transmission, multidisciplinary team, preterm infant

## Abstract

**Aims:**

This study aims to report three cases of maternal-fetal transmission of tuberculosis in premature infants, systematically analyze their clinical characteristics, diagnostic processes, and treatment outcomes, explore the core role of the multidisciplinary team (MDT) in optimizing diagnosis and treatment, and provide an evidence-based basis for early identification, precise diagnosis, and effective therapy to reduce high mortality rates and enhance clinical management levels.

**Methods:**

Through retrospective case reports combined with a literature review, three cases of maternal-fetal transmission of tuberculosis in extremely premature twins and a premature infant were reported. Integration of maternal history, placental pathology assessment (such as acid-fast staining positive bacilli and Mycobacterium tuberculosis qPCR detection), imaging examinations (such as chest x-ray and CT), etiological tests (such as NGS), and fundus examination results. Treatment adopted individualized anti-tuberculosis regimens (isoniazid 10–15 mg/kg/d, rifampicin 15–20 mg/kg/d, pyrazinamide 20–30 mg/kg/d), collaboratively developed by MDT (neonatology, infectious diseases, pharmacists, and ophthalmology experts), combined with supportive therapies (such as mechanical ventilation, blood transfusion, and nutritional support). Follow-up evaluated growth and neurodevelopmental outcomes.

**Results:**

This study reported three cases of maternal-fetal transmission of tuberculosis, involving extremely premature twins at 27^+4^ weeks (birth weights 890 g and 880 g) and one premature infant at 34^+1^ weeks. The twins' mother had a history of tuberculosis of the uterus or uterine TB, fever during pregnancy, and postpartum confirmed tuberculosis (positive placental pathology and NGS); infants presented with respiratory distress and fever. Diagnostic basis included maternal history, placental pathology, NGS detection of Mycobacterium, and fundus examination (retinal white spot changes). Under MDT collaboration, anti-tuberculosis treatment (isoniazid, rifampicin, pyrazinamide) and supportive therapy controlled the infection. The older twin (male) was hospitalized for 93 days, weight reached 3,670 g, and at follow-up to corrected age of 4.5 months, weight increased to 7.44 kg, length 59 cm; the younger twin (female) hospitalized for 60 days, weight 2,170 g, follow-up to corrected age of 4.5 months, weight 6.62 kg, length 54 cm. The twins had normal growth and development, no permanent visual impairment. The premature infant case was similar with good prognosis.

**Conclusion:**

The diagnosis of maternal-fetal transmission of tuberculosis in extremely premature and premature infants is highly challenging due to the lack of specific symptoms, often misdiagnosed as sepsis or respiratory distress syndrome (RDS). This study observed through three cases that integrating chest imaging, etiological tests (such as NGS), placental pathology assessment, and fundus screening (such as retinal white spot changes) aids in early diagnosis. MDT collaboration in developing individualized anti-tuberculosis treatment plans (including isoniazid, rifampicin, and pyrazinamide) supplemented with supportive therapy effectively improves infant prognosis, with normal weight gain post-discharge, good development, and no permanent visual damage. Strengthening prenatal screening and monitoring for high-risk pregnant women helps prevent such cases. In the future, multicenter studies should further optimize diagnostic criteria and treatment strategies to reduce mortality and improve neonatal quality of life.

## 1 Introduction

Tuberculosis (TB) is a major global public health challenge. According to the World Health Organization (WHO) 2024 report, approximately 10.8 million new TB cases (incidence rate of 134 per 100,000 population) were reported worldwide in 2023, with an estimated 217,000 cases among pregnant women (based on the proportion of female cases and the reproductive-age population), primarily concentrated in high-burden countries such as India (26%), Indonesia (10%), China (6.8%), the Philippines (6.8%), and Pakistan (6.3%) (1, 2). Maternal-fetal transmission of TB, encompassing congenital (via placenta or amniotic fluid) and perinatal (during delivery or postpartum contact) infections, is extremely rare (3, 4). Historical literature has reported approximately 400–500 cases of congenital TB, although perinatal infections are more common in high-burden regions (e.g., India, China, and Africa) due to maternal-infant contact and environmental exposure (5–7). However, due to atypical symptoms, limited diagnostic tools, and underreporting, particularly in high TB and HIV burden areas, the actual number of cases may far exceed documented records (6, 8). In high-burden regions, especially those with high HIV prevalence (e.g., Africa), HIV infection increases the risk of active TB in pregnant women, thereby elevating the likelihood of maternal-fetal transmission (9). Untreated congenital TB in newborns carries an extremely high mortality rate of 50%–75%, primarily due to rapid disease progression and the immature neonatal immune system. Even with standard anti-TB treatment, mortality remains at 20%–30%, influenced by diagnostic delays and severe complications such as respiratory failure, multi-organ failure, meningitis, or drug-resistant strains (5, 6, 10).

Extremely preterm infants (gestational age <28 weeks) and preterm infants (gestational age <37 weeks) face a higher risk of tuberculosis due to immature immune and lung functions, and limited metabolic capacity. Their clinical manifestations are insidious (such as fever and respiratory distress) and are easily misdiagnosed as sepsis or pneumonia, thereby significantly increasing the difficulty of diagnosis and treatment (5, 6). Tuberculosis in pregnant women increases the risk of preterm birth by 1.7 times (11). In regions with high TB-HIV comorbidity, such as South Africa, 23% of infants born to mothers with multidrug-resistant or rifampicin-resistant tuberculosis (MDR/RR-TB) develop tuberculosis, and maternal sputum culture positivity at delivery significantly elevates this risk (12).

The clinical features of tuberculosis in extremely preterm infants lack specificity, often presenting as respiratory distress, fever, poor weight gain, or hepatosplenomegaly, which are easily confused with sepsis (3). Diagnosis is limited by low sputum culture positivity rates (<30%), atypical imaging findings, and restrictions on molecular diagnostics (such as Xpert MTB/RIF Ultra) in newborns (3, 13). Treatment requires adjustment of anti-tuberculosis drug dosages to accommodate low body weight and hepatic/renal insufficiency, while being vigilant for drug toxicity (14). MDT collaboration, integrating pediatricians, infectious disease specialists, pharmacists, and public health workers, is key to optimizing management (14). Brazil's experience in significantly reducing mother-to-child transmission of AIDS (with AIDS detection rates in children under 5 years dropping to 1.5/100,000 population in 2022) demonstrates the critical importance of integrating prenatal screening with multidisciplinary comprehensive intervention strategies (15). However, research on the clinical heterogeneity of tuberculosis in extremely preterm infants and MDT applications remains scarce.

This study reports three cases of maternal-fetal transmission of tuberculosis in extremely preterm twins and preterm infants. Combined with a literature review, this study systematically analyzes their clinical features, diagnostic processes, and treatment outcomes, and explores the key role of MDT in case management. The study aims to provide evidence-based support for early identification, precise diagnosis, and effective treatment, to optimize therapeutic strategies, reduce high mortality rates, and to serve as a reference for clinicians to enhance awareness and management capabilities for this rare disease.

## 2 Case reports

### 2.1 Case 1

This male infant, the first twin, was born at a gestational age of 27 ^+^ ^4^ weeks and admitted to our hospital at 46 min of life due to respiratory distress. He was delivered by cesarean section under general anesthesia at the referring hospital on December 30, 2024. Birth weight was 890. Notably, the amniotic fluid was stained with grade III meconium. Apgar scores were 3, 6, and 8 at 1, 5, and 10 min, respectively. Prior to transfer, he received extensive resuscitation at the referring facility, including endotracheal intubation, cardiopulmonary resuscitation (CPR), intratracheal administration of epinephrine and pulmonary surfactant, and positive pressure ventilation via T-piece.

Maternal history: Two years prior to conception, the mother was successfully treated for uterine tuberculosis with a standard anti-tuberculosis regimen. In 2024, she conceived via IVF-ET. From 25 weeks of gestation, she experienced recurrent fever (maximum 40 °C), chest CT indicating bilateral pneumonia, and suspicious positive urinary Mycobacterium tuberculosis DNA, suggesting urinary tract tuberculosis. Anti-tuberculosis treatment was initiated at 25 weeks and 5 days, and symptoms improved after 6 days, leading to self-discharge. At 27 weeks and 4 days, emergency cesarean section was performed due to septic shock, severe pneumonia, respiratory failure, etc. Postpartum temperature was 40 °C, blood oxygen saturation 81% (on 100% oxygen), and she underwent veno-venous extracorporeal membrane oxygenation + continuous renal replacement therapy (VV-ECMO + CRRT). Prenatal examination: Hb 62 g/L, PLT 83 × 10^9^/L, PCT 11.23 ng/mL, IL-2 receptor >7,500 u/mL. Postpartum peripheral blood and bronchoalveolar lavage fluid NGS indicated Mycobacterium tuberculosis infection, placental pathology showed acid-fast staining positive bacilli, and Mycobacterium tuberculosis qPCR detected Mycobacterium tuberculosis DNA (Ct value = 15.71), confirming tuberculosis. The patient received anti-infective therapy with imipenem/cilastatin, caspofungin, tigecycline, colistin, ceftazidime/avibactam, aztreonam, and fluconazole. Concurrently, an individualized anti-tuberculosis regimen was administered, comprising isoniazid, ethambutol, moxifloxacin, linezolid, clofazimine, and cycloserine.

Admission Physical Examination: Tracheal intubation, poor responsiveness, weak spontaneous breathing, coarse breath sounds in both lungs accompanied by coarse wet rales, body temperature 36.5 ℃. Low muscle tone in all four limbs, capillary refill time at the heel 2 s.

Auxiliary Examinations: Routine blood test: WBC 13.7 × 10^9^/L, Hb 119 g/L, PLT 224 × 10^9^/L, neutrophils 72.7%. Liver function: Globulin GLB 13.3 g/L (reference value 18.0–26.0 g/L), hs-CRP 1.7 mg/L (<8 mg/L), PCT 0.67 ng/mL. Chest x-ray suggests NRDS, complicated with neonatal pneumonia. During hospitalization, general microbiological screening revealed a throat swab positive for Ureaplasma urealyticum RNA, while Group B Streptococcus (GBS) screening was negative. Sputum culture indicated coagulase-negative staphylococci. Blood culture, B-type natriuretic peptide (BNP) levels, and cultures from the umbilical venous catheter (UVC) and PICC tips were all negative. Cerebrospinal fluid (CSF) analysis demonstrated an elevated protein level of 1,849.0 mg/L; however, routine tests, smears, and bacterial cultures were otherwise unremarkable. Notably, metagenomic next-generation sequencing (mNGS) of the CSF detected Human metapneumovirus. Given the high risk of disseminated TB in preterm infants, the exclusion of CNS tuberculosis was prioritized. Although CSF protein was elevated (likely attributed to prematurity and viral infection), the specific absence of Mycobacterium tuberculosis sequences in CSF mNGS, combined with negative acid-fast smears and cultures, allowed us to rule out central nervous system involvement. Regarding tuberculosis screening: Repeated gastric aspirates for acid-fast bacilli (collected on Dec 31–Jan 1, Jan 14–16, and Jan 21–23) and multiple Mycobacterium tuberculosis cultures were negative. The T-SPOT.TB assay was indeterminate, while both the Xpert MTB/RIF assay and the purified protein derivative (PPD) skin test yielded negative results.

Discharge Diagnoses: Extremely low birth weight infant (890 g); Extremely preterm infant (GA 27^+4^ weeks); Bronchopulmonary dysplasia (BPD); Neonatal respiratory distress syndrome (NRDS); Neonatal pneumonia; Neonatal asphyxia; Congenital tuberculosis (clinical diagnosis); Perinatal infection; Hemodynamically significant patent ductus arteriosus (hsPDA); Patent foramen ovale (PFO); Neonatal pathological jaundice; Suspected brain injury of prematurity; Anemia of prematurity; Neonatal abdominal distension; Hyperthyrotropinemia; Hypocalcemia; Vitamin D insufficiency; Umbilical hernia; Hemangioma; Retinopathy of prematurity (ROP); Suspected left inguinal hernia; First of twins.

**Figure 1:**
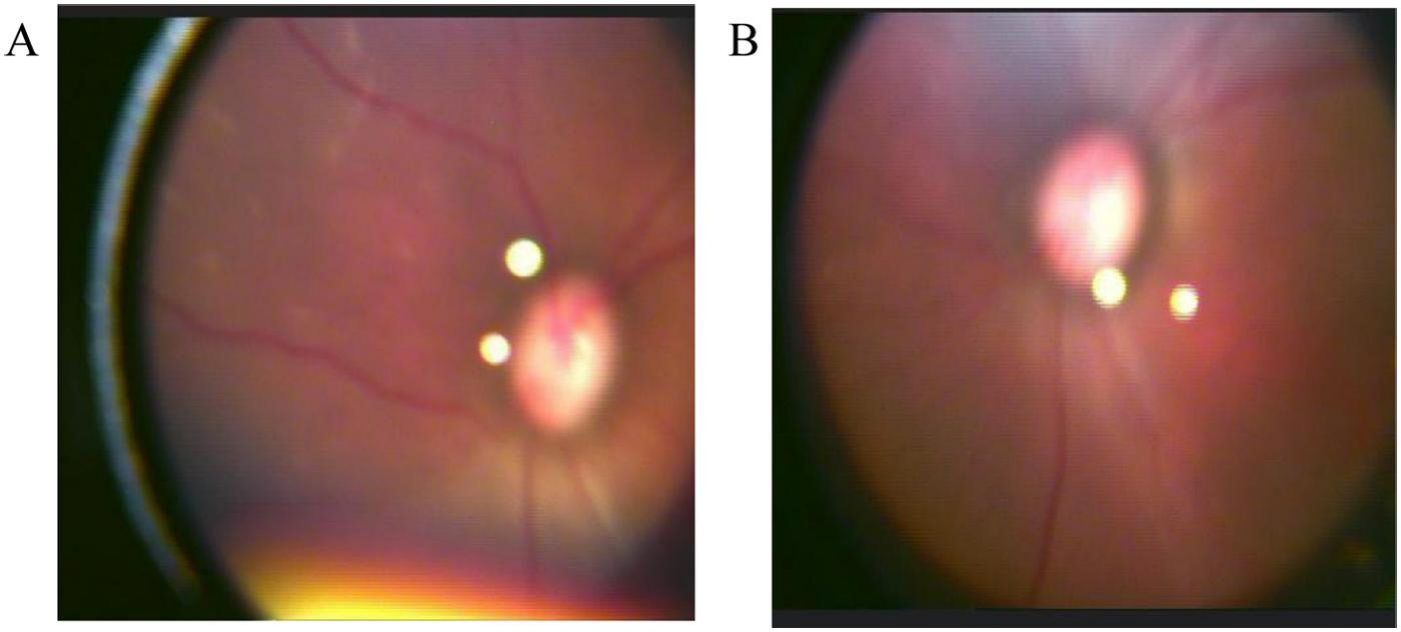
Fundus examination results: white punctate changes observed in the superotemporal quadrant of the retina in the right eye. **(A)** Examination date 2025.03.11 (71 days after birth); **(B)** Examination date 2025.04.01 (92 days after birth).

Treatment and Clinical Course: Upon admission, given the maternal history of septic shock and the infant's critical status (extremely preterm with respiratory distress), there was a high suspicion of severe early-onset sepsis potentially caused by multidrug-resistant organisms. Therefore, adhering to antimicrobial stewardship principles regarding high-risk empiric coverage, initial anti-infective therapy consisted of meropenem combined with ampicillin. This regimen was subsequently de-escalated to cefoperazone-sulbactam after excluding specific multidrug-resistant pathogens, aiming to balance effective infection control with minimizing collateral damage to the microbiome. Azithromycin was administered to treat Ureaplasma urealyticum infection. Additional management included caffeine for respiratory stimulation, red blood cell transfusion for anemia correction (post-transfusion Hb 137 g/L, CRP 3.1 mg/L), and respiratory support via high-frequency/conventional mechanical ventilation and non-invasive BiPAP. Umbilical artery/vein catheterization and PICC placement were performed, along with Vitamin K_1_ administration for bleeding prophylaxis. On the 3rd day after birth, isoniazid 10 mg/(kg·d) and rifampin 15 mg/(kg·d) were given for anti-tuberculosis treatment; pyrazinamide 15 mg/(kg·d) was added on the 15th day, with the regimen being intensive phase of 2 months isoniazid, rifampin, pyrazinamide/maintenance phase of 6–9 months isoniazid, rifampin (2HRZ/6–9HR). From 1 to 5 weeks after birth, manifestations such as cyanosis, shortness of breath, apnea, abdominal distension, jaundice, and edema occurred, treated with citric acid caffeine, vitamin AD, multiple infusions of red blood cell suspension to correct anemia, albumin, diuretics, phototherapy, etc. Fever occurred twice on the 27th day after birth (maximum 37.8 ℃). Due to significant oxygen dependence, BPD was considered, and acetaminophen was given to close the ductus arteriosus, salbutamol nebulization, and dexamethasone to reduce lung inflammation. Transitioned to nasal cannula on the 58th day, intermittent oxygen on the 75th day, and completely off oxygen on the 90th day. Fundus screening (on the 35th and 52nd days after birth) showed bilateral refractive media opacity; on the 71st and 92nd days, it showed white punctate changes in the superior temporal quadrant of the right eye retina (Figures 1A,B), considering the possibility of tuberculosis.

Outcome: The infant was hospitalized for 93 days. At discharge (corrected gestational age 40 weeks and 6 days), the weight was 3,670 g (weight-for-age Z-score: −0.06, based on Fenton 2013 growth chart) and the feeding volume was 65 mL per feed (every 3 h). During hospitalization, the infant completed the intensive phase of treatment with isoniazid, rifampin, and pyrazinamide. Upon discharge, the regimen was switched to the continuation phase with isoniazid and rifampicin, under regular follow-up at the infectious disease outpatient clinic.

### 2.2 Case 2

Female infant, the second twin, was admitted due to “respiratory distress 44 min after premature birth”. She had a gestational age of 27^+4^ weeks and was delivered by cesarean section under general anesthesia at the referring hospital on December 30, 2024. Her birth weight was 880 g, and Apgar scores at 1, 5, and 10 min were 6, 8, and 8, respectively. Post-birth management similar to the first twin.

Auxiliary Examinations: Routine blood test: WBC 9.4 × 10^9^/L, Hb 160 g/L, PLT 334 × 10^9^/L, hs-CRP 2.0 mg/L. Chest x-ray indicated NRDS complicated by potential neonatal pneumonia. Microbiological screening: A throat swab tested positive for Ureaplasma urealyticum RNA (SAT), while GBS screening was negative. Sputum and blood cultures were negative. CSF analysis: Routine tests, biochemistry, smears, and bacterial cultures showed no obvious abnormalities. Notably, mNGS of the CSF was negative. Together with the normal CSF biochemical indices and the absence of specific neurological signs (e.g., seizures or bulging fontanelle), central nervous system tuberculosis was effectively ruled out, justifying the use of the standard congenital tuberculosis regimen rather than the intensified TBM protocol. Regarding tuberculosis screening: Given the dichorionic nature of the twins, specific screening was conducted for this infant. Repeated gastric aspirates for acid-fast bacilli and Mycobacterium tuberculosis cultures were negative. The T-SPOT.TB assay showed no obvious abnormalities, and two separate PPD skin tests yielded negative results. Fundus screening: Performed at 35, 52, 71, and 92 days after birth, examinations revealed bilateral hazy refractive media.

Discharge Diagnoses: Extremely low birth weight infant (880 g); Extremely preterm infant (GA 27^+4^ weeks); BPD; NRDS; Neonatal pneumonia; Neonatal asphyxia; Congenital tuberculosis (clinical diagnosis); Perinatal infection; hsPDA; PFO; Neonatal pathological jaundice; Suspected brain injury of prematurity; Anemia of prematurity; Intracranial hemorrhage (Grade I); Coagulation dysfunction; Hypoalbuminemia; Hyperthyrotropinemia; Hypocalcemia; Vitamin D insufficiency; Second of twins.

Treatment and Clinical Course: After admission, following the same antimicrobial stewardship rationale as for the first twin (high risk of maternal vertical transmission of resistant pathogens), the infant was treated sequentially with meropenem for broad-spectrum empiric coverage, followed by de-escalation to cefoperazone-sulbactam for infection control, alongside azithromycin for Ureaplasma urealyticum. Pharmacological closure of the patent ductus arteriosus was managed with ibuprofen and acetaminophen. Supportive care included caffeine citrate for apnea of prematurity, blood transfusions for anemia, and supplementation with fresh frozen plasma, albumin, thyroxine, and vitamins A and D. Central venous access was established via umbilical catheterization and PICC placement. Anti-tuberculosis treatment followed the same regimen as the first twin. Regarding respiratory support: Invasive ventilation was required for 14 days, followed by non-invasive ventilation for 41 days. The infant was transitioned to intermittent nasal cannula oxygen and successfully weaned off oxygen on day 60.

Outcome: The infant was hospitalized for 60 days. At discharge (corrected gestational age 36 weeks and 1 day), the weight was 2,170 g (weight-for-age Z-score: −0.78, based on Fenton 2013 growth chart) and the feeding volume was 29 mL per feed (every 2 h). Post-discharge, the triple regimen of oral isoniazid, rifampicin, and pyrazinamide was continued for 1 month, followed by the continuation phase with isoniazid and rifampicin, under regular follow-up at the infectious disease outpatient clinic.

Their mother recovered and was discharged after 70 days of inpatient treatment. Follow-up assessments conducted until July 30, 2025 (chronological age: 7 months; corrected age: 4.5 months), confirmed that both twins exhibited normal neurodevelopmental progress and catch-up growth. The first twin (male) weighed 9.0 kg (weight-for-age Z-score: +2.2; length-for-age Z-score: −1.3, based on WHO standards) and measured 62 cm. The second twin (female) weighed 8.1 kg (weight-for-age Z-score: +2.1; length-for-age Z-score: −3.1, based on WHO standards) and measured 57 cm. Both infants continued anti-tuberculosis treatment with isoniazid and rifampicin (continuation phase) and attended regular follow-ups at the infectious disease outpatient clinic, demonstrating favorable growth trends and stable health. Follow-up fundus screening on July 27 revealed normal findings in both infants.

### 2.3 Case 3

Female infant, gestational age 34^+1^ weeks, was admitted 6 h and 25 min post-preterm birth for the management of prematurity. She was delivered vaginally on May 1, 2025, with a birth weight of 1,850 g, and Apgar scores of 10 at 1, 5, and 10 min.

Maternal history: 38 years old, admitted on May 1, 2025, due to “34^+1^ weeks of amenorrhea, abdominal pain for over 1 month, vaginal fluid leakage for half an hour”, resulting in the vaginal delivery of a female infant. The patient denied any history of tuberculosis exposure, reported good mental health during pregnancy, and had normal appetite. On admission, physical examination showed: T 37.8 °C, P 89 beats/min, R 20 breaths/min, BP 124/69 mmHg. Chest CT revealed increased and blurred lung markings, large patchy dense shadows in the lower lobes, diffuse miliary nodules, minimal pericardial effusion, pelvic-abdominal effusion, and cake-like thickening of the greater omentum; abdominal ultrasound indicated a hypoechoic area in the left lower abdomen (depth approximately 7.2 cm); laboratory tests showed a positive Tuberculosis Interferon-Gamma Release Assay (TB-IGRA) in blood (258.68 pg/mL, reference value <0.35 pg/mL), erythrocyte sedimentation rate (ESR) of 120 mm/h; peritoneal puncture fluid was grass-green (acid-fast bacilli negative); placental pathology and qPCR testing were positive for Mycobacterium tuberculosis and the Mycobacterium tuberculosis complex. Consultation with the tuberculosis department suggested a high likelihood of acute hematogenous disseminated pulmonary tuberculosis combined with abdominal tuberculosis. Treatment included isoniazid 0.3 g (in 250 mL of 0.9% sodium chloride injection, intravenous drip, once daily), rifampicin 0.6 g (in 500 mL of 5% glucose injection, intravenous drip, once daily), oral ethambutol 0.75 g (once daily), and pyrazinamide 1.5 g (once daily).

Infant's Auxiliary Examinations: Routine blood test: WBC 14.8 × 10^9^/L, Hb 203 g/L, PLT 303 × 10^9^/L, hs-CRP 1.6 mg/L, PCT 0.31 ng/mL. Chest x-ray suggested potential neonatal pneumonia to be excluded. General microbiological screening including GBS, Ureaplasma urealyticum RNA, sputum culture, and blood culture were all negative. CSF analysis: Routine tests, biochemistry, smears, and bacterial cultures showed no obvious abnormalities. Notably, mNGS of the CSF was negative. Regarding tuberculosis screening: Continuous three-day gastric fluid examinations for acid-fast bacilli were negative. The T-SPOT.TB assay, PPD skin test, and stool Xpert MTB/RIF assay all yielded negative results.

Discharge Diagnoses: Low birth weight infant (1850g); Preterm infant (GA 34^+1^ weeks); Congenital tuberculosis (suspected); Neonatal pathological jaundice.

Treatment and Clinical Course: Upon admission, the infant received supportive care, including thermal management and nutritional support. Phototherapy was administered for neonatal pathological jaundice (bilirubin 6.2 mg/dL). Given the maternal history of active tuberculosis and positive placental findings, a pediatric infectious disease consultation was conducted. Consequently, an individualized anti-tuberculosis regimen was initiated comprising isoniazid (10 mg/kg/d), rifampicin (15 mg/kg/d), and pyrazinamide (35 mg/kg/d). To exclude central nervous system involvement, a lumbar puncture was performed on day 7; CSF analysis and mNGS were negative. This definitive microbiological evidence from the CSF, confirming the absence of Mycobacterium tuberculosis, ruled out CNS tuberculosis and supported the decision to proceed with the standard anti-tuberculosis treatment plan without corticosteroids. Vitamin K1 was also administered. The infant tolerated the medication and feeding well, with stable vital signs and no requirement for respiratory support.

Outcome: The infant was hospitalized for 11 days. At discharge (corrected gestational age 35 weeks and 5 days), the weight was 2,020g (weight-for-age Z-score: −1.35, based on Fenton 2013 growth chart), and the feeding volume was 41 mL per feed (every 3 h). Post-discharge, the oral anti-tuberculosis regimen was continued with isoniazid (0.02 g/dose, once daily), rifampicin (0.03 g/dose, once daily), and pyrazinamide (0.07 g/dose, once daily).

Follow-up: Follow-up assessments conducted until July 30, 2025 (chronological age: 3 months; corrected age: 1.5 months), confirmed that growth and neurodevelopmental progress were within normal ranges. The infant weighed 5.0 kg (weight-for-age Z-score: +0.43, based on WHO standards) and measured 54 cm (length-for-age Z-score: −0.92, based on WHO standards), with a feeding volume of 120 mL per feed (every 2 h). The infant continued anti-tuberculosis treatment (consolidation phase with isoniazid and rifampicin) and attended regular follow-ups at the infectious disease outpatient clinic, demonstrating favorable growth trends and stable health.

## 3 Discussion

### 3.1 Diagnosis and clinical significance of congenital tuberculosis

Congenital tuberculosis (CTB) is a rare but serious disease, primarily transmitted through trans-placental hematogenous spread or aspiration of infectious amniotic fluid. According to a recent systematic review, approximately 92 cases of congenital tuberculosis were reported in China from 1976 to 2018, but due to diagnostic difficulties and underreporting, the actual incidence may be higher, especially in high-burden areas like China (5). This disease has high mortality and morbidity, making early diagnosis and treatment crucial. Cantwell revised the Beitzke (1935) diagnostic criteria in 1994 (16), which remains the primary basis for current clinical diagnosis, including: (1) Confirmed Mycobacterium tuberculosis infection; (2) Meeting any of the following: Lesions from any source appearing in the first week after birth (e.g., skin, lungs, liver); Biopsy or autopsy revealing primary hepatic complex or caseous granulomas; Maternal uterine/genital tuberculosis, or evidence of placental infection (e.g., histological evidence of tubercle bacilli); Exclusion of postnatal exposure to tuberculosis patients. In recent years, studies have emphasized integrating molecular diagnostic techniques, such as qPCR on placental samples, into the Cantwell criteria to improve diagnostic accuracy and sensitivity (17). In this case series, three preterm infants (twins at 27^+4^ weeks gestational age, singleton at 34^+1^ weeks) lacked direct etiological or pathological evidence confirming Mycobacterium tuberculosis infection, but their mothers had active tuberculosis during pregnancy (including endometrial tuberculosis and urinary tuberculosis), combined with positive placental pathology and qPCR detection of Mycobacterium tuberculosis DNA, as well as early respiratory distress in the infants, meeting the “maternal genital or placental infection” condition in the revised Cantwell criteria, thus allowing a definitive diagnosis of congenital tuberculosis.

### 3.2 Clinical manifestations and diagnostic challenges of congenital tuberculosis

Infants with congenital tuberculosis are often preterm or extremely low birth weight, and the clinical manifestations lack specificity, typically onset within a few weeks after birth, but the age of onset varies and may be delayed until several months postpartum (17, 18). Common clinical manifestations include fever, cough, respiratory distress, hepatosplenomegaly, jaundice, growth restriction, cyanosis, poor feeding, abdominal distension, and neurological symptoms such as meningeal irritation signs, with fever, cough, respiratory distress, and hepatosplenomegaly being more common (17, 19, 20). In Case 1, the infant presented with low-grade fever, feeding intolerance, and respiratory distress during hospitalization, which are similar to sepsis, viral infections, or other infectious diseases, making it clinically prone to being overlooked or misdiagnosed.

Traditional diagnostic methods for tuberculosis have certain limitations. Gastric aspirate or sputum smear acid-fast staining microscopy, as a conventional method, is simple to operate with a short turnaround time (usually less than 6 h), but it has low sensitivity in pediatric patients (studies show only 2%–67%), particularly in young children (such as neonates) due to the invasiveness and complexity of sample collection, posing significant challenges; the tuberculin skin test (PPD test) relies on delayed-type hypersensitivity reactions, which typically turn positive several weeks after infection, limiting its value in early diagnosis and inability to distinguish between active and latent tuberculosis; in recent years, molecular techniques such as interferon-*γ* release assays (e.g., T-SPOT) and Xpert MTB/RIF have become common methods for diagnosing pediatric tuberculosis, offering higher sensitivity and faster results to compensate for the shortcomings of traditional methods (17, 21). T-SPOT, as a type of interferon-γ release assay (IGRA), has high sensitivity and specificity, is unaffected by Bacillus Calmette-Guérin (BCG) vaccination, and shows advantages in diagnosing latent tuberculosis infection (LTBI), but it cannot distinguish between active tuberculosis (aTB) and latent tuberculosis, and may increase the risk of false negatives by 20%–30% in immunocompromised states (22). Xpert MTB/RIF uses fully automated real-time polymerase chain reaction technology to detect Mycobacterium tuberculosis DNA, with sensitivity and specificity superior to traditional methods. It is capable of identifying Mycobacterium tuberculosis and rifampicin resistance mutations (such as in the rpoB gene) in a short time (usually about 2 h), significantly improving early diagnostic efficiency, especially suitable for early cases with low bacterial load. However, limitations exist: factors such as extremely low bacterial load may lead to false-negative results, whereas a recent history of tuberculosis may lead to false-positive results due to the persistence of residual genetic material from previous infections (23). In this study, the above-mentioned test results for all three cases were negative. The negative results of immunological assays (e.g., TST and IGRA) may be attributed to the immature immune system in extremely preterm or preterm infants, leading to incomplete T-cell function and an inability to sufficiently produce γ-interferon. Meanwhile, the limitations of microbiological or molecular tests (e.g., Xpert MTB/RIF) are likely due to the paucibacillary nature of the disease, where the extremely low bacterial load in samples increases the risk of false negatives. Therefore, clinical diagnosis should comprehensively evaluate multiple indicators, including clinical symptoms, imaging examinations, etiological evidence, and placental pathological examinations, to ensure accurate judgment.

### 3.3 Treatment strategies and multidisciplinary collaborative management

Younger infants, especially immunocompromised preterm neonates, are at significantly higher risk for miliary and central nervous system (CNS) tuberculosis. Since the treatment for CNS tuberculosis differs significantly from non-neurologic TB (requiring a 4-drug regimen, corticosteroids, and longer duration), ruling out CNS involvement was a critical step in our work-up. In this study, CNS tuberculosis was rigorously excluded in all three cases based on a combination of: (1) lumbar puncture performing CSF analysis; (2) negative CSF mNGS results, which offer higher sensitivity than traditional smears for paucibacillary pediatric TB; and (3) the absence of specific neurological signs or imaging features suggestive of tuberculous meningitis (TBM). This thorough exclusion process ensured that the standard congenital tuberculosis regimen was appropriate. According to the World Health Organization (WHO) 2022 guidelines for the management of tuberculosis in children and adolescents, the standard treatment regimen for neonatal congenital tuberculosis recommends a 2-month intensive phase with triple therapy, including isoniazid (10–15 mg/kg/d), rifampicin (10–20 mg/kg/d), and pyrazinamide (15–30 mg/kg/d), followed by a maintenance phase with isoniazid and rifampicin continued for 4–9 months, with the duration adjusted based on clinical response and severity of lesions (14). This regimen aims to rapidly control the infection while considering the physiological characteristics of newborns, such as immature liver and kidney function and differences in drug metabolism, to minimize the risk of toxicity.

In this study, considering that the infants were extremely preterm or preterm, with extremely low birth weights (<1,000 g), incomplete organ function development, and limimultidisciplinary teamted experience in the use of anti-tuberculosis drugs in such extremely low birth weight preterm infants, the treatment regimen was formulated after consultation by a (MDT, including neonatology, infectious diseases, pharmacists, and respiratory specialists). The MDT comprehensively evaluated factors such as drug distribution in the body, potential hepatorenal toxicity, and neonatal safety, adopting an individualized strategy: initial phase with relatively conservative doses of isoniazid 10 mg/kg/d and rifampicin 15 mg/kg/d, followed by gradual introduction of pyrazinamide 15 mg/kg/d based on tolerance and clinical monitoring, with the overall regimen being an intensive phase of 2HRZ/maintenance phase of 6–9HR (14, 23). This dynamic adjustment process not only ensured the effectiveness of treatment but also reduced the risk of drug-related complications, providing a reproducible clinical template for similar high-risk cases. Multidisciplinary collaboration is crucial in the diagnosis and treatment of congenital tuberculosis, as integrating opinions from multiple experts can optimize regimen design, monitor drug responses, and promptly intervene in potential pitfalls, ultimately improving prognosis and reducing mortality.

### 3.4 Importance of fundus examination in the diagnosis of congenital tuberculosis

The diagnosis and treatment of congenital tuberculosis heavily rely on multidisciplinary collaboration, involving specialties such as obstetrics, neonatology, infectious diseases, ophthalmology, and radiology, to enhance diagnostic accuracy, optimize individualized treatment plans, and significantly improve patient outcomes. In this study, repeated fundus examinations in Case 1 revealed bilateral hazy refractive media and white punctate changes in the superotemporal quadrant of the right retina, suggesting that the tuberculosis infection might have involved ocular structures such as choroiditis or retinitis. These ocular manifestations provide critical auxiliary evidence for diagnosis, particularly when other systemic evidence [e.g., chest x-ray or tuberculin skin test (TST)] is inconclusive or negative, highlighting the clinical value of fundus examination in the diagnosis and management of congenital tuberculosis. It facilitates the early identification of potential ocular lesions (e.g., choroiditis, retinal vasculitis, choroidal nodules, or multifocal choroiditis), guiding the timely initiation of anti-tuberculosis therapy and ocular interventions, thereby reducing the occurrence of long-term sequelae such as permanent visual impairment, retinal scarring, or endophthalmitis (24, 25).

In recent years, research has further confirmed the diagnostic role of fundus examination in pediatric and congenital tuberculosis, particularly through the identification of specific ocular manifestations to aid early diagnosis. Ocular tuberculosis (ocular TB) in neonates and children often presents as posterior uveitis, retinal vasculitis, choroidal nodules, or choroiditis, which can serve as indirect indicators of systemic TB, especially in preterm infants with immature immune function (25, 26). A 2024 prospective study conducted at a tertiary pediatric hospital in North India involving 90 children with systemic tuberculosis showed (27) that papilledema (optic disc edema) was the most common posterior segment manifestation, with an incidence of 22.2%. This ocular sign was frequently observed in children with tuberculous meningitis (58.4% of the study population had tuberculous meningitis, with a significant association with tuberculosis type, *P* < 0.001), and could be detected early through ophthalmologic assessments such as fundus examination to assist in diagnosis and prevent permanent ocular complications and blindness. Additionally, enhanced depth imaging optical coherence tomography (EDI-OCT) serves as a complementary tool to fundus examination, providing more precise visualization of tuberculous lesions, including low-reflective lesions with signal enhancement involving the full thickness of the choroid, high reflectivity in the outer retina (indicating inflammatory infiltration), retinal pigment epithelium (RPE) elevation, and the “sign of contiguity” (localized high-reflective material in the contact area between the choriocapillaris/RPE complex and the overlying neurosensory retina, often accompanied by surrounding subretinal fluid). These features significantly enhance the efficacy of non-invasive monitoring and diagnosis (28). A 2025 narrative review further noted (29) that in TB-endemic regions (where ocular TB incidence ranges from 9%–11%), EDI-OCT can serve as a critical imaging biomarker, particularly for the diagnosis of presumed ocular tuberculosis (OTB). By integrating clinical manifestations, laboratory indicators, and imaging markers, this technique effectively improves differential accuracy from other uveitic conditions such as sarcoidosis, Vogt-Koyanagi-Harada (VKH) disease, or tumors. For instance, tuberculous granulomas typically present as larger, full-thickness low-reflective lesions with high reflectivity in the outer retina, RPE elevation, and the “sign of contiguity”, whereas sarcoid granulomas tend to appear as smaller, partial-thickness choroidal thickenings.

Combining imaging (such as chest CT), etiological testing (such as PCR), and placental pathological evaluation, early fundus examination can enhance diagnostic sensitivity. After standardized treatment, ocular tuberculosis-related manifestations are usually reversible, but if treatment is delayed, it may lead to irreversible visual impairment. In patients with suspected ocular tuberculosis, timely ophthalmological assessment can aid in early diagnosis and effectively manage complications such as retinal vasculitis or macular edema, thereby improving visual function and quality of life (30). For pediatric infectious uveitis (including tuberculosis-related), early diagnosis and intervention can significantly improve visual and overall health prognosis (31). Therefore, for neonates with high-risk factors for congenital tuberculosis, such as a history of maternal-infant transmission, routine fundus screening has become a clinical consensus, which helps to significantly reduce mortality, improve survival rates, and enhance the quality of life for affected children.

## 4 Conclusion

This study reports cases of maternal-infant transmission of tuberculosis in extremely preterm twins and a preterm infant, highlighting the clinical heterogeneity and diagnostic challenges of this disease in neonates. By integrating the mother's tuberculosis history, placental pathological evidence (such as positive acid-fast staining for bacilli and positive qPCR detection of Mycobacterium tuberculosis), imaging examinations (such as chest x-rays indicating NRDS and pneumonia), and fundus examinations (such as white punctate changes in the retina), early diagnosis and timely intervention were achieved. MDT (including neonatology, infectious diseases, pharmacists, and ophthalmology experts) collaboration is central to optimizing management, employing individualized anti-tuberculosis treatment regimens (such as isoniazid, rifampicin, and pyrazinamide, with dose adjustments of 10–15 mg/kg/d), combined with supportive therapies (such as mechanical ventilation, blood transfusion, and nutritional support), which significantly improved the infants' prognosis. These cases emphasize that strengthening prenatal screening and monitoring in high-risk pregnant women (such as those with a history of tuberculosis or infections during pregnancy) can effectively reduce the incidence and transmission of congenital tuberculosis. Overall, although the mortality rate of tuberculosis in preterm infants is high (reaching 50%–75% without treatment), comprehensive strategies such as multidisciplinary collaboration and early fundus screening can significantly reduce the risk of complications, improve survival rates and quality of life, providing valuable references for clinical practice.

## 5 Limitations and future research trends

The limitations of this study primarily include a small sample size (only 3 cases reported), which may lead to insufficient generalizability of clinical features and treatment outcomes; additionally, the diagnosis relied on traditional methods (e.g., sputum culture positivity rate <30%), with limited application of molecular diagnostics (e.g., Xpert MTB/RIF Ultra) in neonates, potentially resulting in missed diagnoses; the follow-up period was short, preventing assessment of long-term neurodevelopmental and visual prognosis; finally, the cases originated from a single institution, lacking multicenter data, which may be influenced by regional and resource biases.

Future research trends should focus on expanding sample sizes through multicenter cohort studies to clarify the epidemiological characteristics and risk factors of maternal-infant transmission of tuberculosis; integrating emerging technologies, such as NGS and EDI-OCT, to improve diagnostic sensitivity and the differential accuracy of ocular manifestations; exploring personalized treatment regimens, including the pharmacokinetics and safety of second-line anti-tuberculosis drugs in extremely low birth weight preterm infants; strengthening prenatal screening protocols for high-risk pregnant women (e.g., combined with HIV co-infection assessment), and evaluating the long-term effectiveness of the MDT model; additionally, through large-sample randomized controlled trials, investigating the value of fundus screening in reducing mortality (targeting <20%) and preventing permanent visual impairment. These trends are expected to further optimize the prevention and control strategies for congenital tuberculosis through interdisciplinary collaboration and technological innovation, reducing the global burden of neonatal TB.

## Data Availability

Due to the sensitive nature of patient data in this case report, the de-identified dataset is not publicly accessible. However, reasonable requests for data access may be directed to the corresponding author for consideration.
